# Supplementary material for: SIO: A Spatioimageomics Pipeline to Identify Prognostic Biomarkers Associated with the Ovarian Tumor Microenvironment
Source: Cancers (Basel). 2021 Apr 8;13(8):1777. doi: 10.3390/cancers13081777 (PMC8068305; doi:10.3390/cancers13081777)
Supplement: Supplementary file 1 [file cancers-13-01777-s001.pdf]

cancers

Supplementary Materials: SIO: A Spatioimageomics Pipeline to Identify Prognostic Biomarkers Associated with the Ovarian Tumor Microenvironment

Ying Zhu, Sammy Ferri-Borgogno, Jianting Sheng, Tsz-Lun Yeung, Jared K. Burks, Paola Cappello, Amir A. Jazaeri, Jae-Hoon Kim, Gwan Hee Han, Michael J. Birrer, Samuel C. Mok and Stephen T.C. Wong

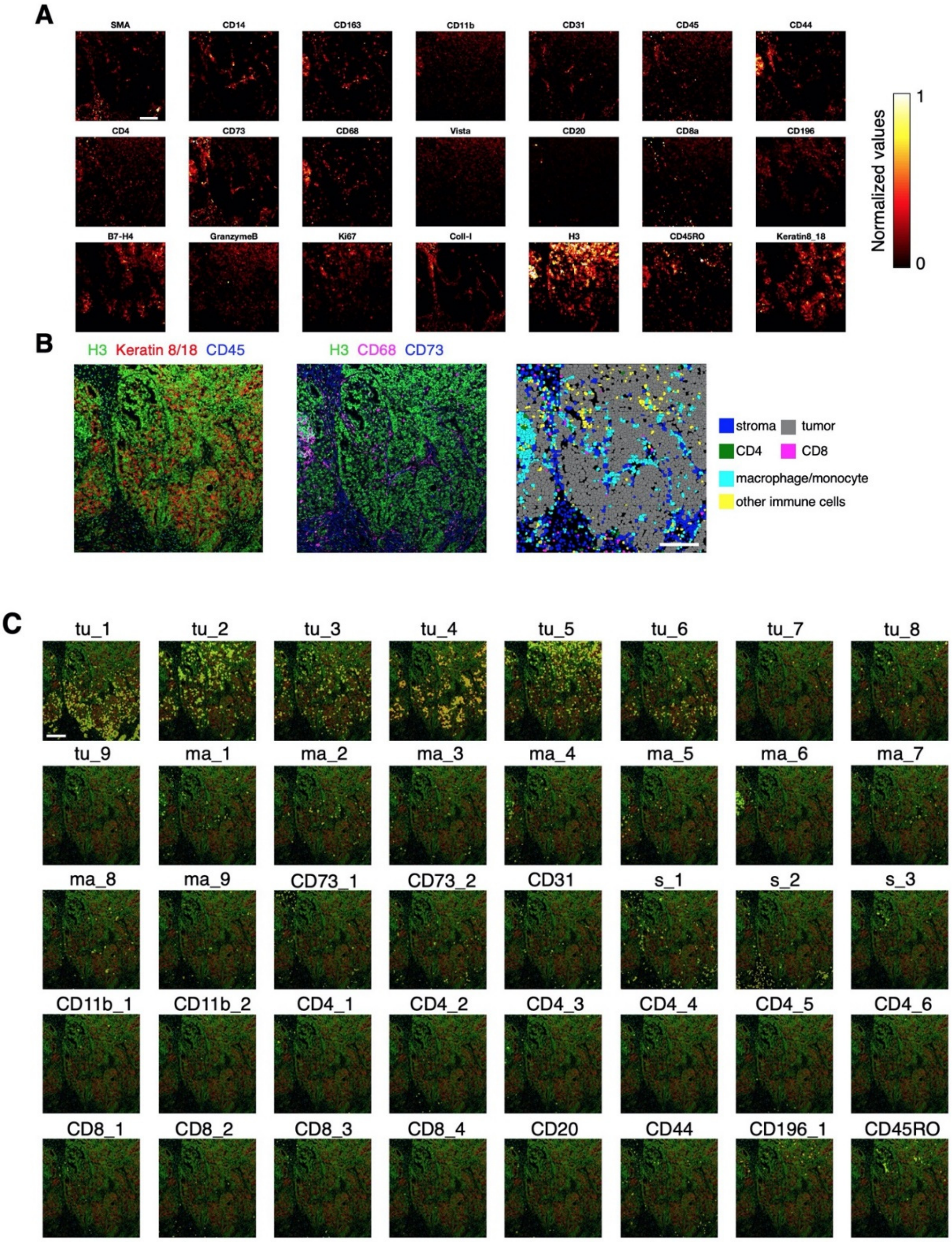

Figure S1. Spatial distribution of all the cell subtypes from a representative sample. **A.** A map of the normalized expression of each marker on the cell masks. **B.** (Left) IMC image with three channels Keratin 8/18, H3, CD45 shown in red, green and blue, respectively. (Middle) IMC image with three channels CD68, H3, CD73 shown in magenta, green and blue, respectively. (Right) The spatial distribution of multiple major cell subtypes. **C**. Spatial distributions of each cell subtype are shown as transparent yellow masks placed on top of the IMC image the same as the one in **B** (left). **A-C** are from the same IMC sample. White scale bar, 200 μm.

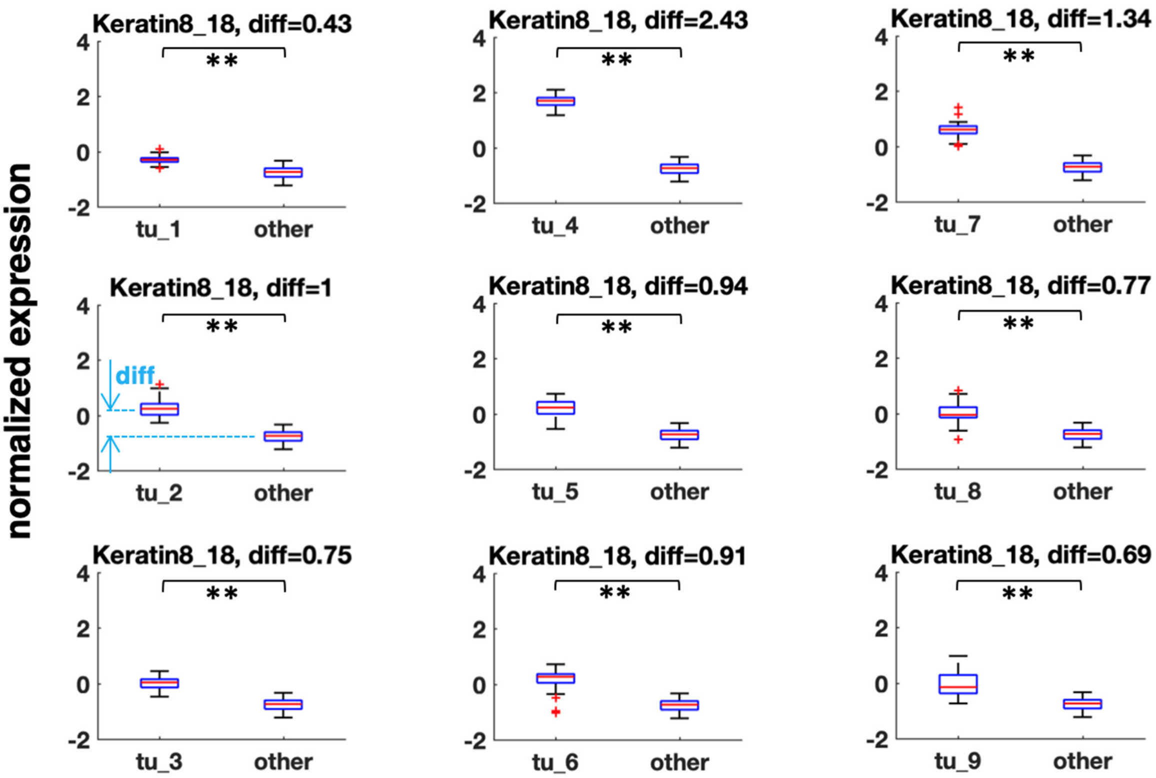

Figure S2. Normalized marker expression of tumor subtypes compared with non-tumor cells. Each data point represents the median of the normalized marker expression of a group of cells for each sample. Two-sample *t* test, $^{**}p < 0.01$. "Diff" is computed as the difference between the mean of two groups.

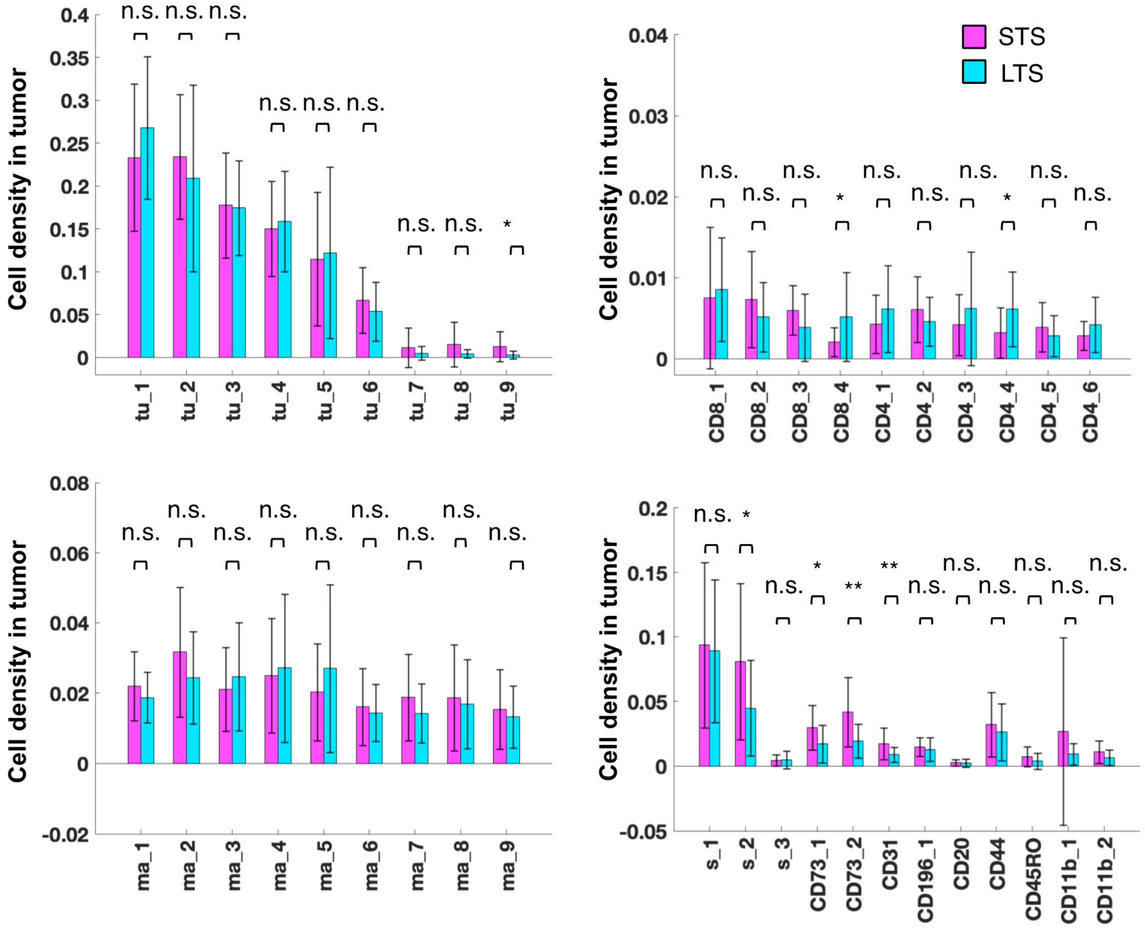

Figure S3. Comparison of cell subtype densities in tumor-enriched regions between long-term survivors (LTS) and short-term survivors (STS). Two sample *t* test, * $p < 0.05$, ** $p < 0.01$, n.s. not significant.

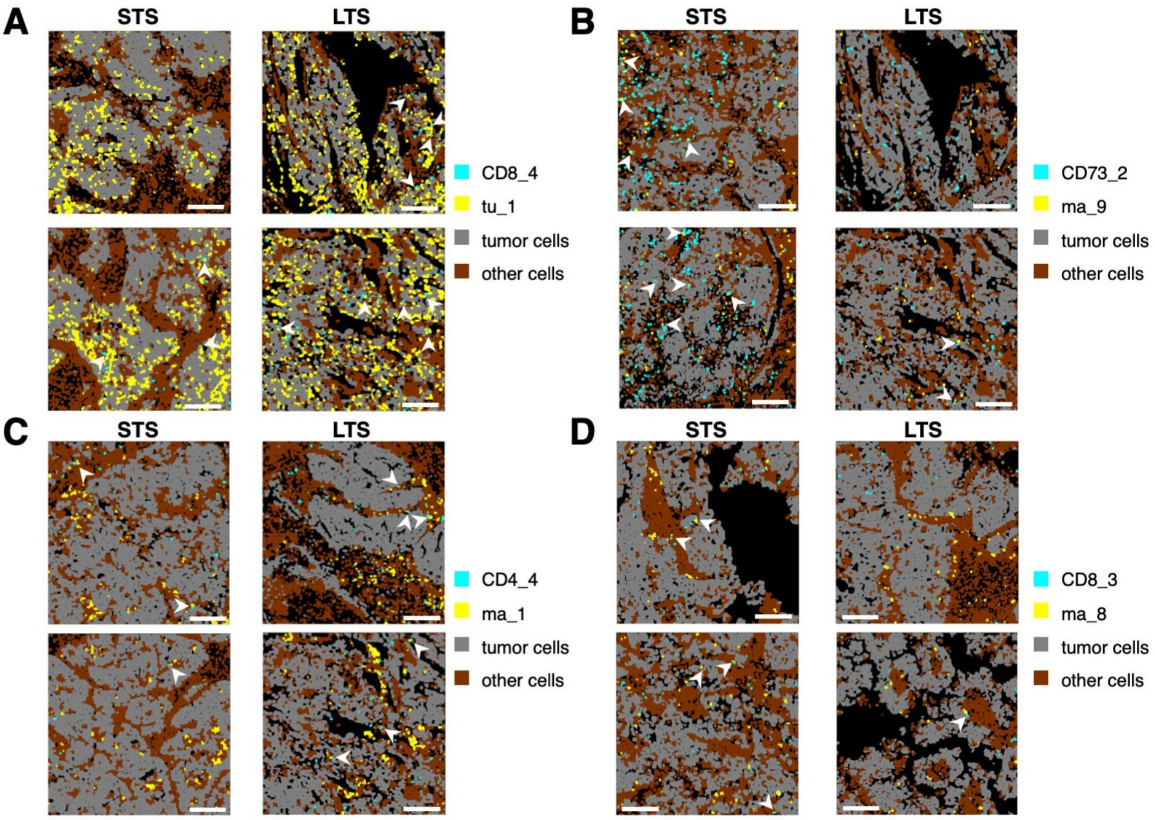

Figure S4. Spatial distribution of pairs of cell subtypes of interest. White scale bar, 200 μm. White arrowhead, interaction between two different cell subtypes. **A.** Interaction between granzyme B^{+} CD8^{+} cytotoxic T cells (CD8_4) and tu_1 tumor cells. **B.** Interaction between CD73^{mid} cells (CD73_2) and CD163^{+} CD68^{+} CD14^{+} macrophages (ma_9). **C.** Interaction between CD45RO^{+} CD4^{+} memory T cells (CD4_4) and CD163^{+} CD68^{+} Vista^{mid} CD14^{+} macrophages (ma_1). **D.** Interaction between CD45RO^{+} CD44^{+} CD8^{+} memory T cells (CD8_3) and CD163^{+} CD14^{mid} macrophages (ma_8) macrophages.

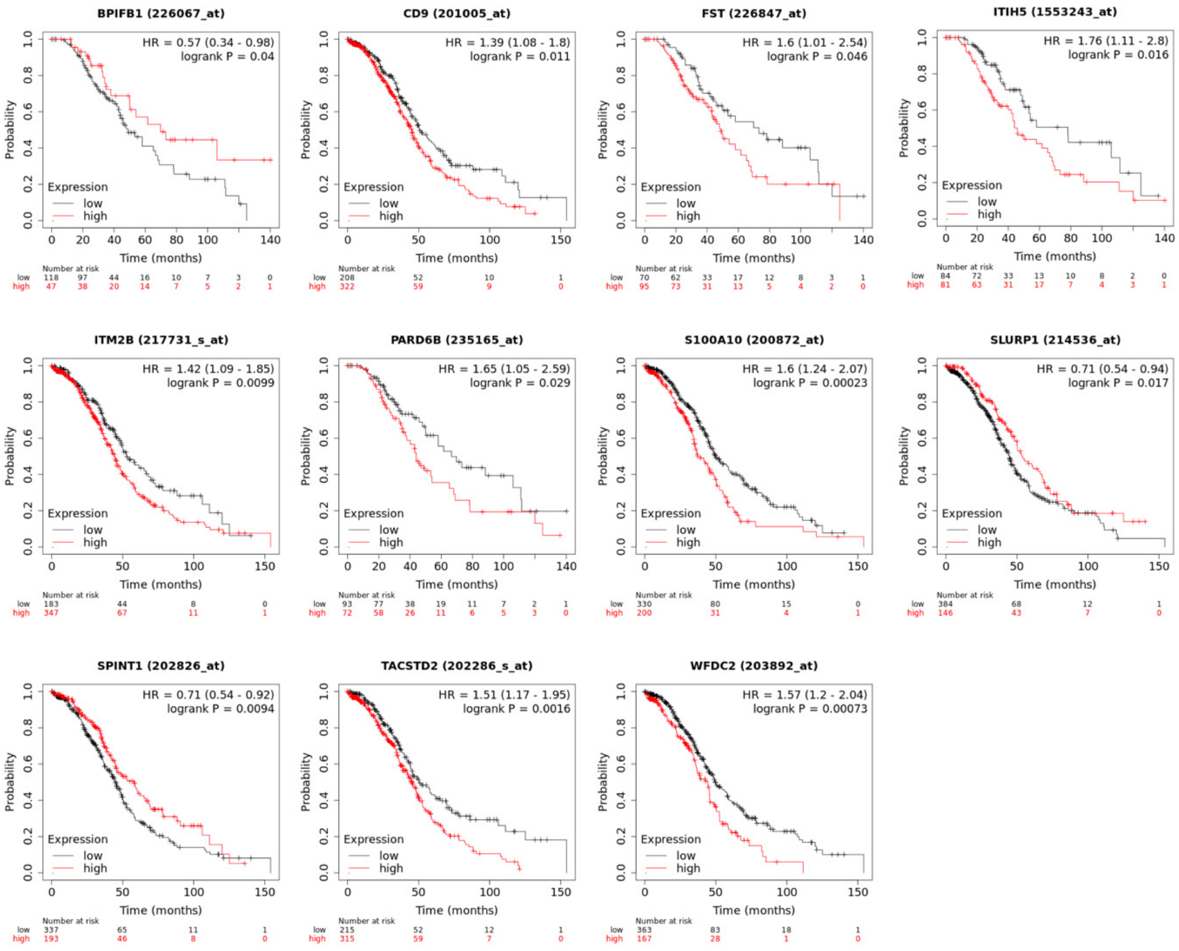

Figure S5. Kaplan-Meier plots of various genes of interest in the microdissected epithelial component of high-grade serous ovarian cancer. HR, hazard ratio.

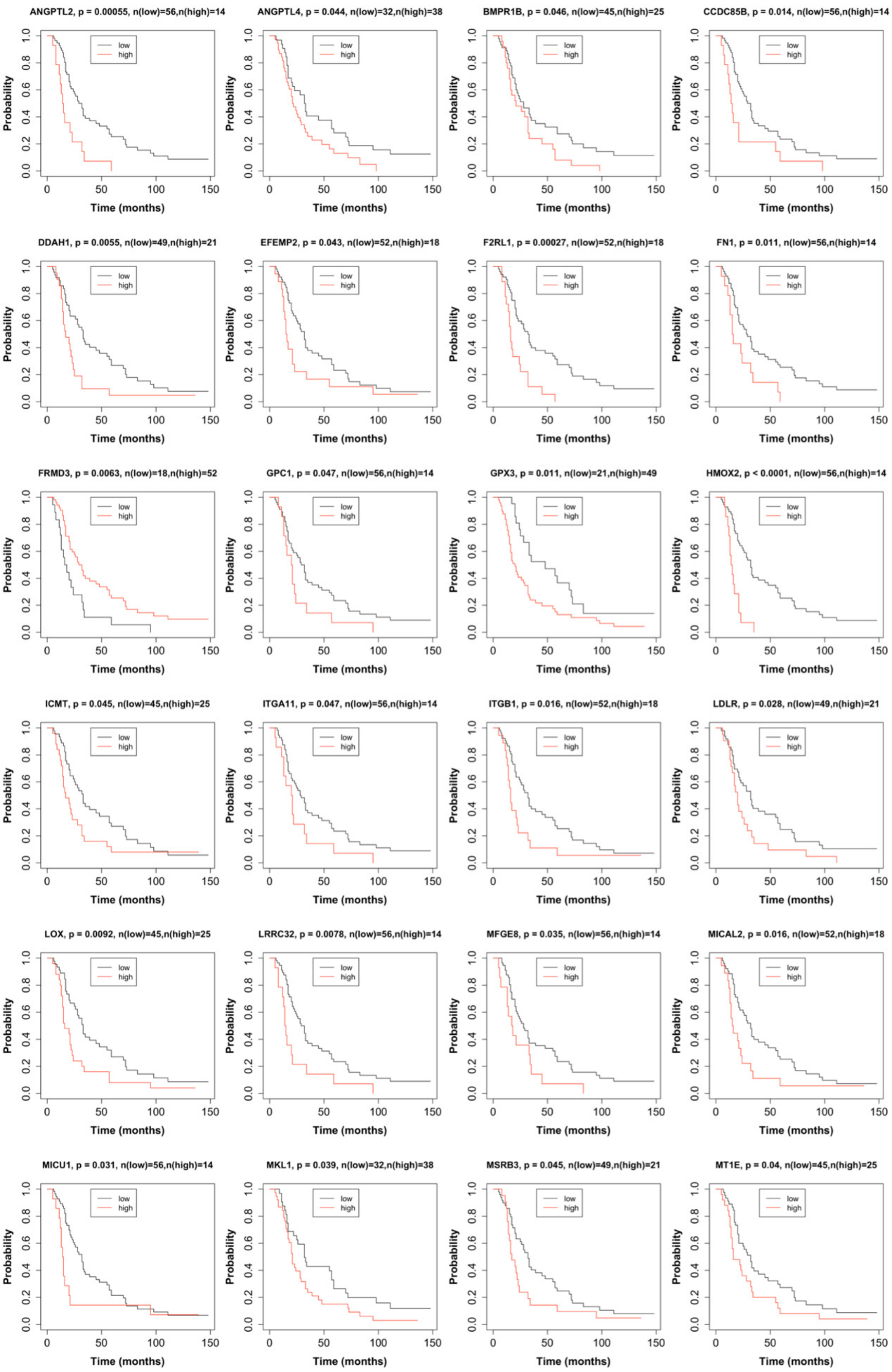
ANGPTL2, p = 0.00055, n(low)=56,n(high)=14
ANGPTL4, p = 0.044, n(low)=32,n(high)=38
BMPR1B, p = 0.046, n(low)=45,n(high)=25
CCDC85B, p = 0.014, n(low)=56,n(high)=14
DDAH1, p = 0.0055, n(low)=49,n(high)=21
EFEMP2, p = 0.043, n(low)=52,n(high)=18
F2RL1, p = 0.00027, n(low)=52,n(high)=18
FN1, p = 0.011, n(low)=56,n(high)=14
FRMD3, p = 0.0063, n(low)=18,n(high)=52
GPC1, p = 0.047, n(low)=56,n(high)=14
GPX3, p = 0.011, n(low)=21,n(high)=49
HMOX2, p < 0.0001, n(low)=56,n(high)=14
ICMT, p = 0.045, n(low)=45,n(high)=25
ITGA11, p = 0.047, n(low)=56,n(high)=14
ITGB1, p = 0.016, n(low)=52,n(high)=18
LDLR, p = 0.028, n(low)=49,n(high)=21
LOX, p = 0.0092, n(low)=45,n(high)=25
LRRC32, p = 0.0078, n(low)=56,n(high)=14
MFGE8, p = 0.035, n(low)=56,n(high)=14
MICAL2, p = 0.016, n(low)=52,n(high)=18
MICU1, p = 0.031, n(low)=56,n(high)=14
MKL1, p = 0.039, n(low)=32,n(high)=38
MSRB3, p = 0.045, n(low)=49,n(high)=21
MT1E, p = 0.04, n(low)=45,n(high)=25
low
high
Probability
Time (months)

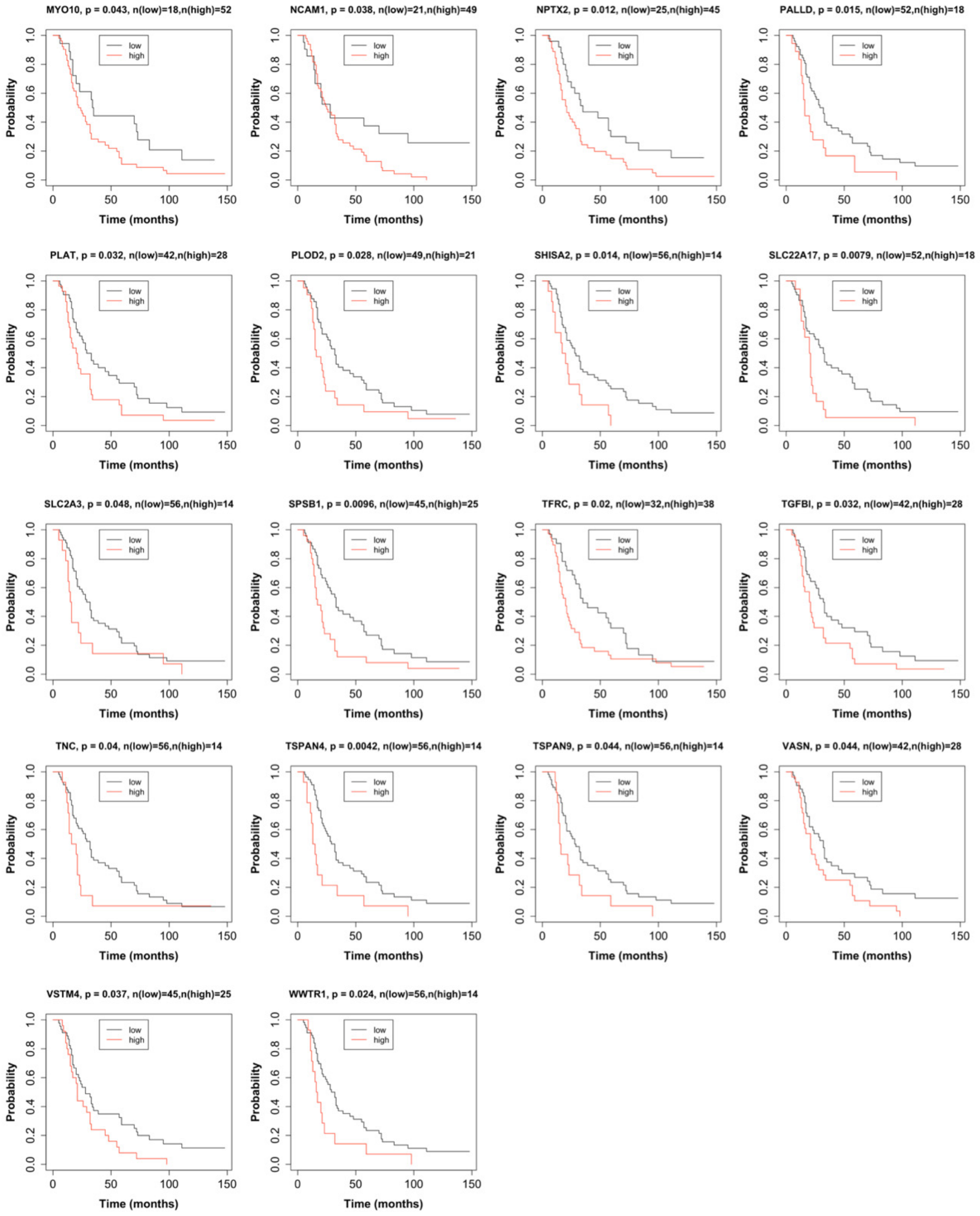

Figure S6. Kaplan-Meier plots of various genes of interest in the microdissected fibroblastic stromal component of high-grade serous ovarian cancer.

Table S1. Clinicopathologic characteristics of samples used in the study.

Variable	LTS	STS	p*
Total number of samples	21	20	
Age			0.013
<50 years	7	2	
>50 years	14	18	
Race			$2.5*10^{-5}$
Asian	16	2	
White	5	18	
Stage			0.61
III	20	18	
IV	1	2	
Grade			0.70
2	5	3	
3	16	17	

LTS, long-term survivors: patients survived ≥60 months, STS, short-term survivors: patients survived ≤20 months, *Fisher exact test (two-sided).

Table S2. Antibodies used for the study.

Metal tag	Antigen	Antibody clone	Catalog *n.*	Vendor
166Er	B7-H4	H74	3166030D	Fluidigm
160Gd	Vista	D1L2G	3160025D	Fluidigm
161Dy	CD20	H1	3161029D	Fluidigm
162Dy	CD8a	D8A8Y	3162035D	Fluidigm
163Dy	CD196/ CCR6	Polyclonal	3163029D	Fluidigm
167Er	Granzyme B	EPR20129-217	3167021D	Fluidigm
168Er	Ki-67	B56	3168022D	Fluidigm
169Tm	Collagen Type I	Polyclonal	3169023D	Fluidigm
171Yb	Histone 3	D1H2	3171022D	Fluidigm
158Gd	CD73	EPR6115	3158031D	Fluidigm
173Yb	CD45RO	UCHL1	3173016D	Fluidigm
174Yb	Keratin 8/18	C51	3174022D	Fluidigm
144Nd	CD14	EPR3653	3144025D	Fluidigm
147Sm	CD163	EDHu-1	3147021D	Fluidigm
149Sm	CD11b	EPR1344	3149028D	Fluidigm
151Eu	CD31	EPR3094	3151025D	Fluidigm
152Sm	CD45	CD45-2B11	3152016D	Fluidigm
156Gd	CD4	EPR6855	3156033D	Fluidigm
153Eu	CD44	IM7	3153029D	Fluidigm
141Pr	SMA	1A4	3141017D	Fluidigm
159Tb	CD68	KP1	3159035D	Fluidigm

Table S3. Spearman correlations between IMC cell densities and selected genes in microdissected epithelial compartment of HGSC.

Gene Name	Correlated IMC Feature	Correlation Coefficient	*p*	Kaplan-Meier Survival *p**	Function	Refs.
BPIFB1	CD4_4	0.533	0.0050	0.04	Inhibits cell migration and invasion; good prognosis	[34]
CD9	CD4_4	−0.434	0.0266	0.011	Cell motility, tumor metastasis, $CD4^+$ T cell activation; poor prognosis in ovarian cancer	[35,36]

FST	CD31	0.493	0.0103	0.046	Regulates autocrine endothelial cell activity and induces angiogenesis	[91,92]
ITIH5	CD8_4<br>CD4_4	−0.442<br>−0.430	0.0237<br>0.0283	0.016	Tumor suppressor gene, extracellular matrix modulator, blocks T cell infiltration	[37]
ITM2B	CD4_4	−0.426	0.0297	0.0099	Induces apoptosis in activated T cells	[86]
PARD6B	CD31	0.446	0.0222	0.029	Increases metastatic potential	[93]
S100A10	CD73_1<br>CD73_2	0.416<br>0.440	0.0342<br>0.0242	0.0002	Involved in tumor progression, invasion, and metastasis; poor prognosis in ovarian cancer	[94]
SLURP1	CD4_4<br>CD73_2	0.451<br>−0.477	0.0206<br>0.0135	0.017	Antitumor activity, regulates $Ca2^+$ signaling following T cell activation	[38,39,56]
SPINT1 (*HAI-1*)	CD31	−0.523	0.0060	0.0094	Regulates metastatic invasion and tumor-immune microenvironment crosstalk; good prognosis	[95,96]
TACSTD2	CD8_4	−0.642	0.0004	0.0016	Epithelial barrier protein, blocks T cell infiltration	[97]
WFDC2 (*HE4*)	CD8_4	−0.529	0.0054	0.0007	Promotes tumor growth, suppresses $CD8^+$ T cell activation; increases chemoresistance in ovarian cancer	[84,85,98,99]

*Kaplan-Meier survival p value shown in Figure S6.

Table S4. Spearman correlations between imaging mass cytometry (IMC) cell densities and selected genes in the micro-dissected fibroblastic stromal compartment of high-grade serous ovarian cancer (HGSC).

**Gene Name**	**Correlated IMC Feature**	**Correlation Coefficient**	***p***	**Kaplan-Meier Survival *p****	**Function**	**Refs.**
ANGPTL2	CD31<br>CD73_1<br>CD73_2	0.461<br>0.558<br>0.672	0.0176<br>0.0030<br>0.0001	0.0005	Induces endothelial cell sprouting; CAF-derived Angptl2 promotes tumor progression	[60,61]
ANGPTL4	CD31	0.441	0.0238	0.044	Increases reactive oxygen species rate favoring tumor growth and decreases immune cell infiltration; poor prognosis	[100]
BMPR1B	CD4_4	−0.508	0.008	0.046	Decreases immune trafficking; poor prognosis	[89]
CCDC85B	CD73_1<br>CD73_2	0.497<br>0.599	0.0097<br>0.0012	0.014	Promotes cancer cell proliferation and invasion	[40]
DDAH1	CD73_2	0.418	0.0332	0.0055	Associated with tumor progression and poor prognosis	[41]

EFEMP2	CD73_1<br>CD73_2	0.556<br>0.589	0.0031<br>0.0015	0.043	Promotes cancer cell growth and invasion; poor prognosis	[42]
F2RL1 (*PAR2*)	CD31<br>CD73_1<br>CD73_2	0.586<br>0.419<br>0.524	0.0016<br>0.0329<br>0.0059	0.0002	Promotes cancer cell invasion and metastasis	[43]
FN1	CD31<br>CD73_1<br>CD73_2	0.425<br>0.733<br>0.548	0.0303<br><0.00010.003<br>7	0.011	Induces cancer and endothelial cell migration	[101,102]
FRMD3	CD73_1	−0.587	0.0016	0.0063	Tumor suppressor	[103]
GPC1	CD73_1<br>CD73_2	0.622<br>0.735	0.0006<br><0.0001	0.047	Associated with tumorigenesis and regulating angiogenesis for cancer progression and invasion	[104]
GPX3	CD4_4<br>CD73_2	−0.403<br>0.444	0.0406<br>0.0230	0.011	Protects cells and enzymes from oxidative damage; poor survival in ovarian cancer	[105]
HMOX2 (*HO-1*)	CD73_1<br>CD73_2	0.416<br>0.554	0.0342<br>0.0032	<0.0001	Activated by the transcription factor Nrf2, increases chemoresistance	[57]
ICMT	CD73_1<br>CD73_2	0.496<br>0.735	0.0098<br><0.0001	0.045	Increases chemoresistance in ovarian cancer	[58]
ITGA11	CD73_1<br>CD73_2	0.675<br>0.652	0.0001<br>0.0003	0.047	Receptor for collagen remodeling and cancer-associated fibroblast migration in the tumor microenvironment	[106]
ITGB1	CD73_2	0.585	0.0016	0.016	Promotes angiogenesis, tumorigenesis, and cancer progression	[44]
LDLR	CD31	0.435	0.0261	0.028	Activates Wnt pathway, involved in cancer progression	[107]
LOX	CD31<br>CD73_1<br>CD73_2	0.436<br>0.718<br>0.611	0.0259<br><0.0001<br>0.0008	0.0092	Driver of tumor migration and angiogenesis; poor prognosis in ovarian cancer	[45,108]
LRRC32 (*GARP*)	CD73_1<br>CD73_2	0.586<br>0.651	0.0016<br>0.0003	0.0078	Regulates TGF-β, which drives cancer-associated fibroblast formation, and inhibits cytotoxicity of $CD8^+$ T cells	[67,88]
MFGE8	CD31<br>CD73_1<br>CD73_2	0.643<br>0.649<br>0.459	0.0003<br>0.0003<br>0.0183	0.035	Increases VEGF and ET-1 levels; enhances M2 polarization of macrophages and promotes tumor angiogenesis	[46,47]
MICAL2	CD31<br>CD73_1<br>CD73_2	0.439<br>0.604<br>0.629	0.0246<br>0.0010<br>0.0005	0.016	Accelerates tumor progression by regulating cell proliferation and epithelial-mesenchymal transition	[48]

MICU1	CD73_1<br>CD73_2	0.558<br>0.676	0.0030<br>0.0001	0.031	Increases chemoresistance; poor survival in ovarian cancer	[109]
MKL1	CD73_1<br>CD73_2	0.598<br>0.613	0.0012<br>0.0008	0.039	Promotes ovarian cancer cell migration and invasion	[110]
MSRB3	CD73_1<br>CD73_2	0.420<br>0.653	0.0323<br>0.0002	0.045	Increases tumorigenesis in ovarian cancer	[49]
MT1E	CD31<br>CD73_1<br>CD73_2	0.459<br>0.586<br>0.587	0.0182<br>0.0016<br>0.0016	0.04	Poor prognosis in ovarian cancer	[111]
MYO10	CD4_4	−0.551	0.0034	0.043	Regulates cancer-associated fibroblast rigidity	[64,65]
NCAM1	CD4_4<br>TU_9	−0.410<br>0.424	0.0374<br>0.0306	0.038	Stimulates ovarian cancer cell migration and invasion	[50]
NPTX2	CD4_4<br>CD73_2	−0.424<br>0.527	0.0306<br>0.0055	0.012	Promotes cancer growth	[51]
PALLD	CD73_1<br>CD73_2	0.631<br>0.715	0.0005<br><0.0001	0.015	Induces activation of stromal fibroblasts, cell migration, and invasion through epithelial-mesenchymal transition	[112]
PLAT (*tPA*)	CD73_1<br>CD73_2	0.507<br>0.643	0.0081<br>0.0003	0.032	Poor prognosis in ovarian cancer	[52]
PLOD2	CD73_1<br>CD73_2	0.636<br>0.544	0.0004<br>0.0040	0.028	Expressed in cancer-associated fibroblasts, promotes tumor cell invasion and migration	[63]
SHISA2	CD73_2	0.535	0.0047	0.014	Regulates WNT5A; enhances cancer aggressive phenotype	[113]
SLC22A17	CD73_1<br>CD73_2	0.458<br>0.422	0.0184<br>0.0313	0.0079	Receptor for LCN2; poor prognosis	[114]
SLC2A3 (*GLUT3*)	CD73_1<br>CD73_2	0.703<br>0.562	<0.0001<br>0.0027	0.048	Promotes tumor cell proliferation; poor prognosis in ovarian cancer	[53,115]
SPSB1	CD73_1<br>CD73_2	0.511<br>0.459	0.0075<br>0.0183	0.0096	Increases proliferation and migration of ovarian cancer cells	[54]
TFRC (*CD71*)	CD73_2	0.400	0.0427	0.02	Enhances the rate of c-Myc-mediated tumor formation	[116,117]
TGFBI	CD73_1<br>CD73_2	0.516<br>0.704	0.0069<br><0.0001	0.032	Produced by tissue-associated macrophages, increases HGSC cell migration	[87]
TNC	CD73_1<br>CD73_2	0.574<br>0.613	0.0021<br>0.0008	0.04	Produced by TAM, increases HGSC cell migration	[69,70,87]
TSPAN4	CD73_1<br>CD73_2	0.533<br>0.559	0.0050<br>0.0029	0.042	Poor prognosis	[118]
TSPAN9	CD73_1<br>CD73_2	0.482<br>0.713	0.0125<br><0.0001	0.044	Chemoresistance; poor prognosis	[59]
VASN	CD73_1<br>CD73_2	0.562<br>0.611	0.0027<br>0.0009	0.044	Stimulates tumor progression and angiogenesis	[55]

VSTM4	CD4_4	−0.429	0.0286	0.037	Negative regulator of T cells	[66]
WWTR1 (*TAZ*)	CD4_4<br>CD73_2	−0.589<br>0.479	0.0015<br>0.0132	0.024	In stromal cells, triggers effects with positive feedback on the growth of tumor cells	[62]

*Kaplan-Meier survival p value shown in Figure 7.

References

91. Janik, S.; Bekos, C.; Hacker, P.; Raunegger, T.; Schiefer, A.-I.; Müllauer, L.; Veraar, C.; Dome, B.; Klepetko, W.; Ankersmit, H.J.; et al. Follistatin impacts Tumor Angiogenesis and Outcome in Thymic Epithelial Tumors. *Sci. Rep.* **2019**, *9*, 1–12, doi:10.1038/s41598-019-53671-8.
92. Kozian, D.H.; Ziche, M.; Augustin, H.G. The activin-binding protein follistatin regulates autocrine endothelial cell activity and induces angiogenesis. *Lab. Investig.* **1997**, *76*, 267–276.
93. E Cunliffe, H.; Jiang, Y.; Fornace, K.M.; Yang, F.; Meltzer, P.S. PAR6B is required for tight junction formation and activated PKCζ localization in breast cancer. *Am. J. Cancer Res.* **2012**, *2*, 478–491.
94. Noye, T.M.; Lokman, N.A.; Oehler, M.K.; Ricciardelli, C. S100A10 and Cancer Hallmarks: Structure, Functions, and its Emerging Role in Ovarian Cancer. *Int. J. Mol. Sci.* **2018**, *19*, 4122.
95. Oberst, M.D.; Johnson, M.D.; Dickson, R.B.; Lin, C.Y.; Singh, B.; Stewart, M.; Williams, A.; al-Nafussi, A.; Smyth, J.F.; and Gabra, H.; et al. Expression of the serine protease matriptase and its inhibitor HAI-1 in epithelial ovarian cancer: Correlation with clinical outcome and tumor clinicopathological parameters. *Clin. Cancer Res. Off. J. Am. Assoc. Cancer Res.* **2002**, *8*, 1101–1107.
96. Gómez-Abenza, E.; Ibáñez-Molero, S.; García-Moreno, D.; Fuentes, I.; Zon, L.I.; Mione, M.C.; Cayuela, M.L.; Gabellini, C.; Mulero, V. Zebrafish modeling reveals that SPINT1 regulates the aggressiveness of skin cutaneous melanoma and its crosstalk with tumor immune microenvironment. *J. Exp. Clin. Cancer Res.* **2019**, *38*, 1–14, doi:10.1186/s13046-019-1389-3.
97. Salerno, E.P.; Bedognetti, D.; Mauldin, I.S.; Deacon, D.H.; Shea, S.M.; Pinczewski, J.; Obeid, J.M.; Coukos, G.; Wang, E.; Gajewski, T.F.; et al. Human melanomas and ovarian cancers overexpressing mechanical barrier molecule genes lack immune signatures and have increased patient mortality risk. *Oncoimmunology* **2016**, *5*, e1240857.
98. Bertin, S.; Lozano-Ruiz, B.; Bachiller, V.; García-Martínez, I.; Herdman, S.; Zapater, P.; Frances, R.; Such, J.; Lee, J.; González-Navajas, J.M.; et al. Dual Specificity Phosphatase 6 (DUSP6) regulates CD4+ T cell functions and restrains the spontaneous colitis in IL-10 deficient mice. *Mucosal Immunol.* **2015**, *8*, 505–515.
99. Hsu, W.-C.; Chen, M.-Y.; Hsu, S.-C.; Huang, L.-R.; Kao, C.-Y.; Cheng, W.-H.; Pan, C.-H.; Wu, M.-S.; Yu, G.-Y.; Hung, M.-S.; et al. DUSP6 mediates T cell receptor-engaged glycolysis and restrains TFH cell differentiation. *Proc. Natl. Acad. Sci. USA* **2018**, *115*, E8027–E8036, doi:10.1073/pnas.1800076115.
100. La Paglia, L.; Listì, A.; Caruso, S.; Amodeo, V.; Passiglia, F.; Bazan, V.; Fanale, D. Potential Role of ANGPTL4 in the Cross Talk between Metabolism and Cancer through PPAR Signaling Pathway. *PPAR Res.* **2017**, *2017*, 1–15, doi:10.1155/2017/8187235.
101. Lou, X.; Han, X.; Jin, C.; Tian, W.; Yu, W.; Ding, D.; Cheng, L.; Huang, B.; Jiang, H.; Lin, B. SOX2 Targets Fibronectin 1 to Promote Cell Migration and Invasion in Ovarian Cancer: New Molecular Leads for Therapeutic Intervention. *OMICS A J. Integr. Biol.* **2013**, *17*, 510–518, doi:10.1089/omi.2013.0058.
102. Zou, L.; Cao, S.; Kang, N.; Huebert, R.C.; Shah, V.H. Fibronectin Induces Endothelial Cell Migration through β1 Integrin and Src-dependent Phosphorylation of Fibroblast Growth Factor Receptor-1 at Tyrosines 653/654 and 766. *J. Biol. Chem.* **2012**, *287*, 7190–7202.
103. Haase, D.; Meister, M.; Muley, T.; Hess, J.; Teurich, S.; Schnabel, P.; Hartenstein, B.; Angel, P. FRMD3, a novel putative tumour suppressor in NSCLC. *Oncogene* **2007**, *26*, 4464–4468, doi:10.1038/sj.onc.1210225.
104. Wang, S.; Qiu, Y.; Bai, B. The Expression, Regulation, and Biomarker Potential of Glypican-1 in Cancer. *Front. Oncol.* **2019**, *9*, 614, doi:10.3389/fonc.2019.00614.
105. Worley, B.L.; et al. GPx3 supports ovarian cancer progression by manipulating the extracellular redox environment. *Redox Biol.* **2019**, *25*, 101051.
106. Zeltz, C.; Alam, J.; Liu, H.; Erusappan, P.M.; Hoschuetzky, H.; Molven, A.; Parajuli, H.; Cukierman, E.; Costea, D.-E.; Lu, N.; et al. $\alpha 11\beta 1$ Integrin is Induced in a Subset of Cancer-Associated Fibroblasts in Desmoplastic Tumor Stroma and Mediates In Vitro Cell Migration. *Cancers* **2019**, *11*, 765, doi:10.3390/cancers11060765.
107. Roslan, Z.; Muhamad, M.; Selvaratnam, L.; Ab-Rahim, S. The Roles of Low-Density Lipoprotein Receptor-Related Proteins 5, 6, and 8 in Cancer: A Review. *J. Oncol.* **2019**, *2019*, 1–6, doi:10.1155/2019/4536302.
108. Baker, A.-M.; Bird, D.; Welti, J.C.; Gourlaouen, M.; Lang, G.; Murray, G.I.; Reynolds, A.R.; Cox, T.R.; Erler, J.T. Lysyl Oxidase Plays a Critical Role in Endothelial Cell Stimulation to Drive Tumor Angiogenesis. *Cancer Res.* **2013**, *73*, 583–594, doi:10.1158/0008-5472.can-12-2447.
109. Chakraborty, P.K.; Mustafi, S.B.; Xiong, X.; Dwivedi, S.K.D.; Nesin, V.; Saha, S.; Zhang, M.; Dhanasekaran, D.; Jayaraman, M.; Mukherjee, P.; et al. MICU1 drives glycolysis and chemoresistance in ovarian cancer. *Nat. Commun.* **2017**, *8*, 14634.
110. Xu, W.; Xu, H.; Fang, M.; Wu, X.; Xu, Y. MKL1 links epigenetic activation of MMP2 to ovarian cancer cell migration and invasion. *Biochem. Biophys. Res. Commun.* **2017**, *487*, 500–508, doi:10.1016/j.bbrc.2017.04.006.
111. Si, M.; Lang, J. The roles of metallothioneins in carcinogenesis. *J. Hematol. Oncol.* **2018**, *11*, 1–20, doi:10.1186/s13045-018-0645-x.

112. Brentnall, T.A.; Lai, L.A.; Coleman, J.; Bronner, M.P.; Pan, S.; Chen, R. Arousal of Cancer-Associated Stroma: Overexpression of Palladin Activates Fibroblasts to Promote Tumor Invasion. *PLoS ONE* **2012**, *7*, e30219.
113. Tamura, K.; Furihata, M.; Satake, H.; Hashida, H.; Kawada, C.; Osakabe, H.; Fukuhara, H.; Kumagai, N.; Iiyama, T.; Shuin, T.; et al. SHISA2 enhances the aggressive phenotype in prostate cancer through the regulation of WNT5A expression. *Oncol. Lett.* **2017**, *14*, 6650–6658, doi:10.3892/ol.2017.7099.
114. Miyamoto, T.; Kashima, H.; Yamada, Y.; Kobara, H.; Asaka, R.; Ando, H.; Shiozawa, T. Lipocalin 2 Enhances Migration and Resistance against Cisplatin in Endometrial Carcinoma Cells. *PLoS ONE* **2016**, *11*, e0155220, doi:10.1371/journal.pone.0155220.
115. Masin, M.; Vazquez, J.; Rossi, S.; Groeneveld, S.; Samson, N.; Schwalie, P.C.; Deplancke, B.; Frawley, L.E.; Gouttenoire, J.; Moradpour, D.; et al. GLUT3 is induced during epithelial-mesenchymal transition and promotes tumor cell proliferation in non-small cell lung cancer. *Cancer Metab.* **2014**, *2*, 11, doi:10.1186/2049-3002-2-11.
116. O'Donnell, K.A.; Yu, D.; Zeller, K.I.; Kim, J.-W.; Racke, F.; Thomas-Tikhonenko, A.; Dang, C.V. Activation of Transferrin Receptor 1 by c-Myc Enhances Cellular Proliferation and Tumorigenesis. *Mol. Cell. Biol.* **2006**, *26*, 2373–2386, doi:10.1128/mcb.26.6.2373-2386.2006.
117. Shen, Y.; Li, X.; Dong, D.; Zhang, B.; Xue, Y.; Shang, P. Transferrin receptor 1 in cancer: A new sight for cancer therapy. *Am. J. Cancer Res.* **2018**, *8*, 916–931.
118. Qi, W.; Sun, L.; Liu, N.; Zhao, S.; Lv, J.; Qiu, W. Tetraspanin family identified as the central genes detected in gastric cancer using bioinformatics analysis. *Mol. Med. Rep.* **2018**, *18*, 3599–3610, doi:10.3892/mmr.2018.9360.
